# A survey of Australian rheumatologists’ perspectives of nutrition needs in systemic sclerosis

**DOI:** 10.1177/23971983231185465

**Published:** 2023-07-06

**Authors:** De-arne A Samm, Aimee R Macoustra, Rhiannon K Crane, Leah M McWilliams, Susanna M Proudman, Lee-anne S Chapple

**Affiliations:** 1 Department of Nutrition and Dietetics, Royal Adelaide Hospital, Adelaide, SA, Australia; 2 Department of Nutrition and Dietetics, Central Adelaide Local Health Network, Royal Adelaide Hospital, Adelaide, SA, Australia; 3 Rheumatology Unit, Royal Adelaide Hospital, Adelaide, SA, Australia; 4 Discipline of Medicine, The University of Adelaide, Adelaide, SA, Australia; 5 Intensive Care Unit, Royal Adelaide Hospital, Adelaide, SA, Australia; 6 Adelaide Medical School, The University of Adelaide, Adelaide, SA, Australia

**Keywords:** Systemic sclerosis, rheumatology, gastrointestinal symptoms, service delivery, dietetics, nutrition

## Abstract

**Aim:**

Systemic sclerosis (scleroderma) is an incurable inflammatory condition synonymous with unique nutrition needs. As rheumatologists are frequently responsible for managing the various organ manifestations, this study aimed to understand the service needs and nutritional concerns of rheumatologists involved in the care of adults with systemic sclerosis.

**Methods:**

A 13-item online qualitative and quantitative survey was distributed via REDCap^®^ from January to March 2022 to rheumatologists who are members of the Australian Scleroderma Interest Group and consult patients with systemic sclerosis. Data were collected on rheumatologists’ demographics, and their views on symptoms observed, nutrition concerns and priorities, and preferred dietetic service provision for their patients. Data are reported as number (%).

**Results:**

Of 27 eligible rheumatologists, 17 (63%) completed the survey. All rheumatologists reported gastrointestinal symptoms in their patients (*n* = 17, 100%); predominantly reflux (*n* = 17, 100%) and dysphagia (*n* = 17, 100%). Weight loss was observed by the majority of rheumatologists (*n* = 15, 88%). Rheumatologists reported patients used food avoidance/special diets to manage symptoms (*n* = 12, 71%). Dietetic consultation was reported as potentially beneficial by all rheumatologists, with the preferred time being when symptoms increase or change (*n* = 15, 88%), and the preferred approaches being written resources (*n* = 15, 88%), face-to-face (*n* = 14, 82%) and telephone consultation (*n* = 14, 82%). Advice on gaining weight (*n* = 14, 82%) and systemic sclerosis symptom management (*n* = 13, 77%) were the most desired education topics reported.

**Conclusion:**

Rheumatologists commonly observe gastrointestinal symptoms in patients with systemic sclerosis and report dietetics services would be advantageous in supporting their patients to gain weight and better manage their symptoms.

## Introduction

Systemic sclerosis (SSc), also known as scleroderma, is an autoimmune disease associated with multiorgan involvement, with excessive collagen production leading to hardening of internal and external structures.^1^ As there is no cure for SSc, treatment is focused on symptom management.^2^ While fibrosis of the skin is the characteristic organ involved and may impact meal preparation and self-feeding, approximately 90% of patients will also experience symptoms of gastrointestinal (GI) tract involvement.^3,4^ These symptoms are diverse, with both upper (e.g. gastroesophageal reflux disease, gastric dysmotility, dysphagia) and lower (e.g. diarrhoea, constipation) involvement, with severity increasing with disease progression.^5,6^

As a result of these symptoms, patients with SSc are susceptible to nutritional decline. Up to 56% of patients with SSc are considered at high risk of malnutrition,^3,7–9^ likely impacted by inadequate dietary intake or impaired nutrient absorption.^10^ Malnutrition has been associated with an increased risk of mortality,^11^ and reduced quality of life in other populations.^12^ Given the frequency of nutrition-impacting symptoms and increased risk of malnutrition, nutrition intervention is thought central to SSc management.

As symptom manifestation in SSc is heterogeneous,^13^ individual management strategies are likely required to optimise health outcomes. The ability to access knowledgeable healthcare providers and resources for symptom management has been highlighted as a key challenge for patients with SSc.^4^ Furthermore, a systematic review of clinical practice guidelines for the diagnosis, monitoring, and treatment of patients with SSc reported that no guideline addresses the contribution of specific healthcare providers on SSc management.^14^ While the UK Scleroderma Study Group Consensus Best Practice pathway does highlight the importance of dietetic input in managing specific symptoms,^15^ there are no studies to our knowledge that define optimal dietetic service models for these patients. As one of the first-line healthcare providers for this condition, and in the absence of specialised dietetics advice, rheumatologists are likely to be primarily responsible for managing nutrition education for these patients. Therefore, the aims of this survey were to (1) understand the perceived nutritional concerns of rheumatologists involved in the outpatient care of adults with SSc across Australia and (2) to identify potential dietetic service models for implementation.

## Materials and methods

### Study design

A 13-item online quantitative survey was developed to identify nutritional concerns and priorities of rheumatologists for this population and preferred dietetic service delivery models to inform future service plans (Supplemental File S1). Ethics and governance approvals were obtained from the Central Adelaide Local Health Network. An information sheet was embedded into the online survey and completion of the survey inferred consent.

### Participant population

Eligible participants included rheumatologists who were members of the Australian Scleroderma Interest Group (ASIG) and working within an outpatient service for SSc within Australia. A total of 27 rheumatologists were eligible for study involvement. Given this small number, a survey completion rate of 50% was considered a priori to be appropriate.

### Survey dissemination

The survey was distributed from 1 February to 10 March 2022 by the Chair of ASIG as an investigator on the study. Eligible participants were contacted through distribution of the survey link via email to the ASIG member list. Reminder emails were sent on 15 February and 10 March 2022.

### Survey design

The web-based survey was designed using Research Electronic Data Capture (REDCap®) software. The survey comprised of two participant demographic questions, six questions about symptoms and management for patients with SSc, and five questions about preferred nutrition education needs. Pilot testing of the survey was conducted from 9 to 16 December 2021, with feedback provided on overall flow, question interpretation, and appropriateness of included questions. This also informed the estimated survey completion time.

### Statistical analyses

Following survey closure, data were downloaded from the REDCap^®^ server into an EXCEL^®^ spreadsheet to facilitate analysis. Standardised descriptive statistics included frequency (number (*n*)) and percentage (%) for categorical variables.

## Results

Among the 27 eligible participants, 17 (63%) completed the survey. The majority of rheumatologists worked in Victoria (*n* = 10, 63%) and spent <1 day per week managing patients with SSc (*n* = 9, 53%; Table 1).

**Table 1.** Participant characteristics.

Variable	Number (%)
Geographical location: (*n* = 16)	
South Australia	2 (13)
Victoria	10 (63)
Tasmania	1 (6)
Western Australia	2 (13)
New South Wales	1 (6)
Time spent working in scleroderma clinic: (*n* = 17)	
⩾5 days a week	1 (6)
3–4 days a week	3 (18)
1–2 days a week	4 (24)
<1 day a week	9 (53)

### Symptoms of SSc

**Figure 1.**
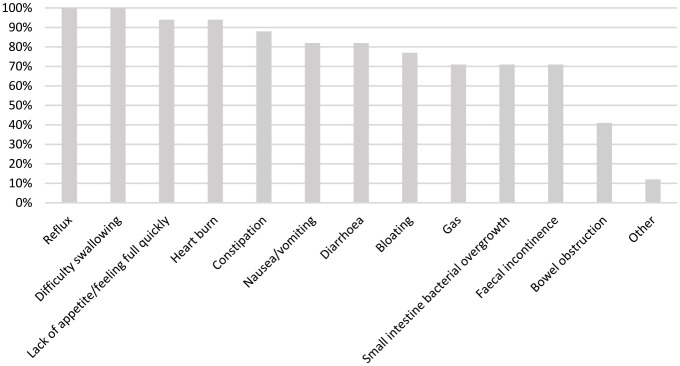
Reported gastrointestinal symptoms observed in patients with systemic sclerosis.

Among those surveyed, 17 (100%) reported seeing patients with SSc-related symptoms (i.e. arthritis, contractures, ulcers, pain, GI issues). The prevalence of these symptoms is reported in Table 2. All rheumatologists reported seeing GI symptoms in their patients (*n* = 17, 100%), which rheumatologists reported to occur ‘often’ (*n* = 12, 71%). The most predominant GI symptoms reported were reflux and difficulty swallowing (both *n* = 17, 100%; Figure 1).

**Table 2.** Prevalence of symptoms of systemic sclerosis observed by rheumatologists.

Variable	Number (%)
Arthritis	
Never	0 (0)
Rarely	0 (0)
Sometimes	8 (47)
Often	8 (47)
Always	1 (6)
Contractures	
Never	0 (0)
Rarely	1 (6)
Sometimes	7 (41)
Often	9 (53)
Always	9 (53)
Ulcers on hands/feet	
Never	0 (0)
Rarely	0 (0)
Sometimes	3 (81)
Often	13 (81)
Always	0 (0.0)
Pain/soreness	
Never	0 (0.0)
Rarely	0 (0.0)
Sometimes	2 (12)
Often	15 (88)
Always	0 (0.0)
Gastrointestinal issues	
Never	0 (0.0)
Rarely	0 (0.0)
Sometimes	2 (12)
Often	12 (71)
Always	3 (18)
Other	
Never	0 (0.0)
Rarely	0 (0.0)
Sometimes	3 (20.0)
Often	10 (67)
Always	2 (13)

### Weight loss from SSc

All rheumatologists surveyed reported seeing weight loss in their patients, with the frequency of this reported as ‘often’ (*n* = 4, 24%), ‘sometimes’ (*n* = 11, 65%), or ‘rarely’ (*n* = 2, 4%).

### Supportive therapies

Three quarters of rheumatologists (*n* = 12, 71%) reported that their patients avoided specific foods or followed special diets to alleviate symptoms (Table 3). A third of the rheumatologists reported their patients used a low FODMAP diet (*n* = 6, 35%) and five reported patients use strategies to aid swallowing (avoid dry foods/soft diet; 29%). Only two rheumatologists (12%) reported that their patients needed support for shopping or cooking.

**Table 3.** Special diets followed by patients with systemic sclerosis as reported by rheumatologists.

Variable	Number (%)
Food avoidance/special diets followed	12 (71)
Low FODMAP diet	6 (50)
Avoid dry foods/soft diet	5 (42)
Foods that ‘lower reflux’ e.g. spicy	3 (25)
Avoid foods that trigger symptoms	3 (25)
No meat	2 (17)
Gluten free diet	2 (17)
Oral nutrition support	1 (8)

FODMAP: Fermentable oligosaccharides, disaccharides, monosaccharides, and polyols.

### Nutrition education needs

The most common nutrition education topic rheumatologists thought their patients required was advice on how to gain weight (*n* = 14, 82%), followed by managing nutrition-related SSc symptoms (*n* = 13, 76%), and special diet information (e.g. low FODMAP, texture modified diet) (*n* = 10, 59%; Table 4). Dietetic consultation was reported as beneficial by all rheumatologists, with the preferred time being when symptoms increase or change (*n* = 15, 88%), followed by consultation at the time of diagnosis (*n* = 7, 41%) and twice yearly (*n* = 5, 29%). The preferred consultation methods were written resources (*n* = 15, 88%), followed by face-to-face and telephone consultation (both *n* = 14, 82%). All rheumatologists thought it would be beneficial to receive education about nutrition for SSc themselves.

**Table 4.** Types of nutrition information frequently required by patients with systemic sclerosis and methods of delivery reported by rheumatologists.

Variable	Number (%)
Topics of education	
Gaining weight	14 (82)
Managing nutrition-related scleroderma symptoms	13 (77)
Special diet information (e.g. texture modified, FODMAPs)	10 (59)
Losing weight	5 (29)
General food knowledge	4 (24)
Managing food intolerances/allergies	0 (0)
Cooking techniques	0 (0)
Other (to specify)	2 (12)
Methods of education delivery	
Written resources	15 (88)
Face to face consultations	14 (82)
Telephone consultations	14 (82)
Group education sessions	11 (65)
Telehealth	11 (65)
Online webinars/YouTube videos	9 (53)
Podcasts	7 (41)
Dietitians in GP clinics	7 (41)
Student-led clinics	3 (18)
Other (will specify)	0 (0)
Frequency of dietetic input	
I do not foresee the need for nutrition education at this time	0 (0)
At diagnosis	7 (41)
Fortnightly	0 (0)
Every month	1 (6)
Twice yearly	5 (29)
Whenever symptoms occur/increase/change	15 (88)
My patients do not need face-to-face consultation but access to online resources all the time would be useful	0 (0)

FODMAP: Fermentable oligosaccharides, disaccharides, monosaccharides, and polyols; GP: general practitioner.

## Discussion

This is one of the first surveys to our knowledge to quantify Australian rheumatologists’ nutrition needs and preferred dietetic service models when managing patients with SSc. Rheumatologists reported observing SSc-related symptoms in their patients, predominantly GI symptoms. Most rheumatologists had observed dietary modifications in their patients including the use of a low FODMAP diet and the avoidance of dry foods/soft diets. All rheumatologists reported that dietetic services would benefit their patients, with written resources being the preferred service delivery mode.

In our survey, all rheumatologists reported observing nutrition-impacting symptoms in their patients with SSc. GI-related symptoms were the most common, predominantly those related to oesophageal motility (reflux and difficulty swallowing). These results align with observational data that report GI symptoms to be the most common experienced symptom by patients with SSc. In a survey of over 400 patients with SSc, 94% of patients reported experiencing upper and 79% lower GI symptoms.^16^ Furthermore, a systematic review of studies on GI dysmotility in SSc patients reported that oesophageal dysmotility was the most commonly occurring GI motility disorder in these patients.^17^ This highlights a key area of intervention for these patients.

The majority of rheumatologists completing our survey reported their patients experienced GI symptoms ‘often’, with 20% of responses stating they see GI symptoms ‘always’. These data are supported by a UK-based study in 402 patients where GI symptoms were reported to occur daily in 10% of the patients that responded.^16^ Further to this, the impact of GI symptoms on quality of life has been previously reported, suggestive of a high severity and/or frequency. In a qualitative survey of Canadian patients with SSc, GI symptoms – including difficulty swallowing and diarrhoea – were reported to ‘sometimes’ or ’moderately’ impact quality of life in more than 80% of patients.^18^ Similarly, focus groups with patients with SSc concluded that difficulty swallowing, digestion, constipation, diarrhoea and bloating affected participants’ lifestyle.^19^ Given the prevalence and frequency of occurrence of GI symptoms, and the potential impact these may have on nutrition, early management of these complications is vital in order to manage the long-term consequences of SSc.

Rheumatologists in our survey reported that patients were using specific diets or dietary restrictions to manage different aspects of their SSc symptoms. However, high-quality evidence for dietary restriction in this patient population is scarce. This is supported by a 2019 systematic review of clinical practice guidelines for SSc that concluded few studies addressed the impact of a dietary intervention on GI symptoms, with limited evidence to support dietary restriction in these patients.^14^ Observational data provide some insight into the role of dietary modification in patients with SSc. In a prospective observational study, self-imposed dietary restrictions to manage GI symptoms – such as avoiding night-time eating to manage reflux – were found to be effective.^20^ Similarly, an observational study found 40% of patients experience fructose malabsorption that could be effectively managed with dietetic-guided dietary restriction (low FODMAP diet).^21^ However, given self-imposed dietary restrictions may exacerbate weight loss and malnutrition and lead to further nutrient deficiencies in an already high-risk population,^22,23^ the role of special diets in these patients requires careful management. Further work should investigate the efficacy of these specific dietary modifications in patients with SSc.

In our survey, all rheumatologists who responded felt that dietetic consultation would be beneficial to the patients they treated with SSc. While few studies explore the impact of a dietetic-led intervention on patient outcomes for SSc, the potential role in general symptom management has been highlighted. Both clinical guidelines and an expert panel document support referring patients at risk of malnutrition to a dietitian.^24,25^ An 18-patient pilot study assessed the impact of a 6-week dietitian-led medical nutrition therapy (MNT) intervention in addition to usual medical management, addressing calorie and protein intake, modified textures, and lifestyle modifications, demonstrating reduced symptom burden and sarcopenia prevalence; however, as a pilot trial only, outcomes were not powered.^22^ Furthermore, a 2022 systematic literature review demonstrated that artificial nutrition can be beneficial in SSc, including oral nutrition support, enteral nutrition, and parenteral nutrition, all of which require implementation by a dietitian.^26^ While further work is required to ascertain optimal dietetic interventions for patients with SSc, healthcare services should consider assessing patients for nutritional risk using a validated screening tool and providing access to dietetic services for symptom management where required.

The optimal dietetic service model identified by rheumatologists in our survey included written resources, face-to-face and telephone consultations being the preferred methods of contact. To the best of our knowledge, no randomised trial has compared dietetic service models in this population. The type of nutrition information accessed by patients with SSc has been quantified previously through focus group discussions. Only 58% of participants had accessed a healthcare professional (including alternative or complementary therapists), while 85% used print media and 77% accessed web-based or social media platforms.^27^ This indicates the need for diversity in resource provision in order to provide highly accessible and credible nutrition information for patients with SSc.

In our survey, all rheumatologists felt they would benefit from receiving education about nutrition for SSc. While rheumatologists have a comprehensive understanding of the complex needs of patients with SSc, they are likely to have competing needs, with the risk that nutrition management may be of a lower priority than other aspects of care as seen in other areas of clinical nutrition.^28^ The lack of specialised training in nutrition for medical professionals has been highlighted previously: a US survey of medical students reported an average of just 19.6 contact hours of nutrition education throughout their entire medical course.^29^ This demonstrates the need to improve nutrition education for health professionals, particularly for rheumatologists without access to a dietitian within their health service.

Our study had a high completion rate with more than half of the target population participating in the survey. However, this survey was only open to Australian rheumatologists who were members of ASIG which may have excluded views of other rheumatologists within Australia who work with patients with SSc, and limits generalisability to other geographical regions. Furthermore, given the small number of rheumatologists in the ASIG email distribution list, our data may have been positively skewed towards those clinicians who had an interest in nutrition, and hence spent time completing the survey. Another limitation of our survey is that questions did not ask rheumatologists to quantify the number or percentage of patients they observed to have specific symptoms, rather asking them to report if they encountered a particular symptom. The survey also did not ask about current dietetics services available within SSc outpatient clinics, reasons for weight changes or percentage of weight loss observed, access to other medical specialities for symptom management such as gastroenterology, or the use of nutritional risk screening tools to identify patients that may benefit from nutrition intervention.

## Conclusion

Rheumatologists commonly observe GI symptoms in patients with SSc, and these symptoms occur often. Rheumatologists report dietetic services would be advantageous in supporting their patients to gain weight and better manage their symptoms.

## Supplemental Material

sj-pdf-1-jso-10.1177_23971983231185465 – Supplemental material for A survey of Australian rheumatologists’ perspectives of nutrition needs in systemic sclerosis Click here for additional data file. Supplemental material, sj-pdf-1-jso-10.1177_23971983231185465 for A survey of Australian rheumatologists’ perspectives of nutrition needs in systemic sclerosis by De-arne A Samm, Aimee R Macoustra, Rhiannon K Crane, Leah M McWilliams, Susanna M Proudman and Lee-anne S Chapple in Journal of Scleroderma and Related Disorders

## References

[1] Odonwodo A Badri T Hariz A. Scleroderma. Treasure Island, FL: StatPearls, 2022.

[2] Shah AA Wigley FM. My approach to the treatment of scleroderma. Mayo Clin Proc 2013; 88(4): 377–393. 23541012 10.1016/j.mayocp.2013.01.018 PMC3666163

[3] Harrison E Herrick AL McLaughlin JT , et al. Malnutrition in systemic sclerosis. Rheumatology 2012; 51(10): 1747–1756. 22850183 10.1093/rheumatology/kes160

[4] Milette K Thombs BD Maiorino K , et al. Challenges and strategies for coping with scleroderma: implications for a scleroderma-specific self-management program. Disabil Rehabil 2019; 41(21): 2506–2515. 29741963 10.1080/09638288.2018.1470263

[5] Nagaraja V McMahan ZH Getzug T , et al. Management of gastrointestinal involvement in scleroderma. Curr Treatm Opt Rheumatol 2015; 1(1): 82–105. 26005632 10.1007/s40674-014-0005-0 PMC4437639

[6] Sobolewski P Maślińska M Wieczorek M , et al. Systemic sclerosis – multidisciplinary disease: clinical features and treatment. Reumatologia 2019; 57(4): 221–233. 31548749 10.5114/reum.2019.87619 PMC6753596

[7] Hvas CL Harrison E Eriksen MK , et al. Nutritional status and predictors of weight loss in patients with systemic sclerosis. Clin Nutr ESPEN 2020; 40: 164–170. 33183531 10.1016/j.clnesp.2020.09.030

[8] Caimmi C Caramaschi P Venturini A , et al. Malnutrition and sarcopenia in a large cohort of patients with systemic sclerosis. Clin Rheumatol 2018; 37(4): 987–997. 29196890 10.1007/s10067-017-3932-y

[9] Caporali R Caccialanza R Bonino C , et al. Disease-related malnutrition in outpatients with systemic sclerosis. Clin Nutr 2012; 31(5): 666–671. 22417677 10.1016/j.clnu.2012.02.010

[10] Abu-Shakra M Guillemin F Lee P. Gastrointestinal manifestations of systemic sclerosis. Semin Arthritis Rheum 1994; 24(1): 29–39. 10.1016/0049-0172(94)90097-3 7985035

[11] Krause L Becker MO Brueckner CS , et al. Nutritional status as marker for disease activity and severity predicting mortality in patients with systemic sclerosis. Ann Rheum Dis 2010; 69(11): 1951–1957. 20511612 10.1136/ard.2009.123273

[12] Rios TC de Oliveira LPM da Costa MLV , et al. A poorer nutritional status impacts quality of life in a sample population of elderly cancer patients. Health Qual Life Outcomes 2021; 19(1): 90. 33731093 10.1186/s12955-021-01735-7 PMC7968230

[13] Barnett AJ Miller MH Littlejohn GO. A survival study of patients with scleroderma diagnosed over 30 years (1953-1983): the value of a simple cutaneous classification in the early stages of the disease. J Rheumatol 1988; 15(2): 276–283. 3361537

[14] Smith V Scirè CA Talarico R , et al. Systemic sclerosis: state of the art on clinical practice guidelines. RMD Open 2018; 4(Suppl. 1): e000782. 10.1136/rmdopen-2018-000782 PMC6203100 30402270

[15] Hansi N Thoua N Carulli M , et al. Consensus best practice pathway of the UK scleroderma study group: gastrointestinal manifestations of systemic sclerosis. Clin Exp Rheumatol 2014; 32(6 Suppl. 86): S214–S221. 25372804

[16] Thoua NM Bunce C Brough G , et al. Assessment of gastrointestinal symptoms in patients with systemic sclerosis in a UK tertiary referral centre. Rheumatology 2010; 49(9): 1770–1775. 20530510 10.1093/rheumatology/keq147

[17] Sallam H McNearney TA Chen JD. Systematic review: pathophysiology and management of gastrointestinal dysmotility in systemic sclerosis (scleroderma). Aliment Pharmacol Ther 2006; 23(6): 691–712. 16556171 10.1111/j.1365-2036.2006.02804.x

[18] Bassel M Hudson M Taillefer SS , et al. Frequency and impact of symptoms experienced by patients with systemic sclerosis: results from a Canadian National Survey. Rheumatology 2011; 50(4): 762–767. 21149249 10.1093/rheumatology/keq310

[19] Suarez-Almazor ME Kallen MA Roundtree AK , et al. Disease and symptom burden in systemic sclerosis: a patient perspective. J Rheumatol 2007; 34(8): 1718–1726. 17611983

[20] Burlui AM Cardoneanu A Macovei LA , et al. Diet in scleroderma: is there a need for intervention? Diagnostics 2021; 11(11): 2118. 34829464 10.3390/diagnostics11112118 PMC8620611

[21] Marie I Leroi AM Gourcerol G , et al. Fructose malabsorption in systemic sclerosis. Medicine 2015; 94(39): e1601. 10.1097/MD.0000000000001601 PMC4616824 26426642

[22] Doerfler B Allen TS Southwood C , et al. Medical nutrition therapy for patients with advanced systemic sclerosis (MNT PASS): a pilot intervention study. JPEN J Parenter Enteral Nutr 2017; 41(4): 678–684. 26209221 10.1177/0148607115597883

[23] Hill P Muir JG Gibson PR. Controversies and recent developments of the low-FODMAP diet. Gastroenterol Hepatol 2017; 13(1): 36–45. PMC5390324 28420945

[24] Baron M Bernier P Côté LF , et al. Screening and therapy for malnutrition and related gastro-intestinal disorders in systemic sclerosis: recommendations of a North American expert panel. Clin Exp Rheumatol 2010; 28(2 Suppl. 58): S42–S46. 20576213

[25] Bharadwaj S Tandon P Gohel T , et al. Gastrointestinal manifestations, malnutrition, and role of enteral and parenteral nutrition in patients with scleroderma. J Clin Gastroenterol 2015; 49(7): 559–564. 25992813 10.1097/MCG.0000000000000334

[26] Keane N Ghannam A Fragkos KC , et al. Oral, enteral and parenteral nutritional therapies in scleroderma: a systematic review. Clin Nutr ESPEN 2022; 51: 174–184. 36184202 10.1016/j.clnesp.2022.06.108

[27] Østbø N Jimenez EY Harb S , et al. Nutrition information resources used by people with systemic sclerosis and perceived advantages and disadvantages: a nominal group technique study. ACR Open Rheumatol 2021; 3(8): 540–549. 34196508 10.1002/acr2.11293 PMC8363851

[28] Chapple LS Chapman M Shalit N , et al. Barriers to nutrition intervention for patients with a traumatic brain injury: views and attitudes of medical and nursing practitioners in the acute care setting. JPEN J Parenter Enteral Nutr 2017; 42(2): 318–326. 29443400 10.1177/0148607116687498

[29] Adams KM Kohlmeier M Zeisel SH. Nutrition education in U.S. medical schools: latest update of a national survey. Acad Med 2010; 85(9): 1537–1542. 20736683 10.1097/ACM.0b013e3181eab71b PMC4042309

