# Supplementary material for: A survey of Australian rheumatologists’ perspectives of nutrition needs in systemic sclerosis
Source: J Scleroderma Relat Disord. 2023 Jul 6;8(3):203–9. doi: 10.1177/23971983231185465 (PMC10515988; doi:10.1177/23971983231185465)
Supplement: sj-pdf-1-jso-10.1177_23971983231185465 – Supplemental material for A survey of Australian rheumatologists’ perspectives of nutrition needs in systemic sclerosis [file sj-pdf-1-jso-10.1177_23971983231185465.pdf]

VENUS- Health Professional

You are invited to complete the VENUS survey below.

You have been selected because you are a rheumatologist who provides care to patients living with Scleroderma and are a member of the Australian Scleroderma Interest Group (ASIG). Your experience will provide valuable information to improve our understanding of how dietetics may improve health outcomes in patients with Scleroderma.

Your survey responses will be known only to the investigators directly involved in the study conduct. Once the survey has closed, your data will be de-identified before it is analysed. Completion of the survey will be considered as implied consent of involvement.

Please find more information in the Participant Information Sheet below.

The survey includes 3 demographics questions, 4 questions on your management of Scleroderma, and 5 questions on possible methods of nutrition education in the future.

It should take 10-15 minutes to complete.

For further information or any queries regarding the survey, please contact the following:

De-arne Samm | Clinical Dietitian

Royal Adelaide Hospital Mobile: 0466 617 264 Phone: (08)7074 0000 pager 1338 Email: de-arne.samm@sa.gov.au

---

[Attachment: "The VENUS Survey Participant Information Sheet - Medical Professionals V2 28.10.21 clean.docx"]

PERSONAL INFORMATION

1. Please state what hospital or health facility you work at: ______________________________

2. Please state how much of your time is dedicated to caring for patients with scleroderma?
   - ◯ ≥5 days a week
   - ◯ 3-4 days a week
   - ◯ 1-2 days a week
   - ◯ < 1 day a week

MANAGING PATIENTS WITH SCLERODERMA

3. In your opinion, please circle how often you see the below symptoms in patients with scleroderma:

	never	rarely	sometimes	often	always
Arthritis	◯	◯	◯	◯	◯
Contractures	◯	◯	◯	◯	◯
Ulcers on hands/feet	◯	◯	◯	◯	◯
Pain/soreness	◯	◯	◯	◯	◯
Gastrointestinal issues	◯	◯	◯	◯	◯
Other symptoms	◯	◯	◯	◯	◯

Do you often see patients with pain in the following areas of their body?	☐ Hands<br>☐ Face<br>☐ Feet<br>☐ Hips<br>☐ Knees<br>☐ Shoulders<br>☐ Back
Do you often see patients with any of the following gastrointestinal issues?	☐ Reflux<br>☐ Difficulty swallowing<br>☐ Nausea/vomiting<br>☐ Lack of appetite/feeling full quickly<br>☐ Heartburn<br>☐ Constipation<br>☐ Gas<br>☐ Diarrhoea<br>☐ Bloating<br>☐ Small Intestine Bacterial Overgrowth<br>☐ Bowel obstruction<br>☐ Faecal incontinence<br>☐ Other
What other GI issues do you often see in patients?	______________________
What other symptoms of scleroderma do you see? Please specify.	______________________
4. How often do you observe weight loss in the Scleroderma patients you treat?	◯ Never<br>◯ Rarely<br>◯ Sometimes<br>◯ Often<br>◯ Always
5. Do most of your patients require any support for cooking and/or shopping?	◯ Yes<br>◯ No
5a. If yes, please specify what support they require for cooking/shopping:	______________________
5b. What are the common reasons for your patients requiring support with shopping/cooking?	______________________
6. Do you regularly see patients avoid any foods and/or follow a special diet because of scleroderma?	◯ Yes<br>◯ No
6a. If yes, please specify which foods/special diet (e.g. gluten free diet, dairy free diet, FODMAP diet, etc):	______________________

NUTRITION EDUCATION NEEDS

7. Please tick the following boxes corresponding to the type of nutrition information most frequently required by patients with scleroderma in your experience:	☐ Gaining weight<br>☐ Losing weight<br>☐ Managing nutrition related scleroderma symptoms<br>☐ Special diet information (e.g. texture modified diet or FODMAP diet)<br>☐ Managing food intolerances/allergies<br>☐ Cooking techniques<br>☐ General food knowledge<br>☐ Other
7a. What other types of nutrition information do you think are frequently required by patients with scleroderma?	__________________________________
8. Do you think a dietitian consult/education would be of benefit to your patients with scleroderma?	◯ Yes<br>◯ No
8a. What mode/s do you believe would be suitable to deliver dietary advice by a dietitian to patients with scleroderma?	☐ Face to face consultations<br>☐ Telephone consultations<br>☐ Group education sessions<br>☐ Written resources i.e., pamphlets, leaflets, booklets etc<br>☐ Online webinars/YouTube videos<br>☐ Podcasts<br>☐ Telehealth<br>☐ Student-led clinics<br>☐ Dietitians in GP clinics<br>☐ Other
8b. Are there any other modes of nutrition education that may be beneficial to your patients with scleroderma?	__________________________________
9. How confident would you feel for University students to provide nutrition education for scleroderma patients (as facilitated by a supervisor)? Please tick:	◯ Not confident at all<br>◯ Slightly confident<br>◯ Fairly confident<br>◯ Completely confident<br>◯ Don't know
10. How often do you believe it is necessary for nutrition education/support to be given to patients with scleroderma?	☐ At diagnosis<br>☐ Fortnightly<br>☐ Every month<br>☐ Twice yearly<br>☐ Whenever symptoms occur/increase/change<br>☐ My patients do not need a face to face consultation but access to online resources all the time would be useful<br>☐ I do not forsee the need for nutrition education at this time
11. Would you as the health professional find it beneficial to receive education about nutrition for patients with scleroderma?	◯ Yes<br>◯ No
12. Thank you for completing the survey. Please use this space to provide any other comments or questions you may have regarding the survey or upcoming research:	__________________________________
